# Tea consumption and risk of bladder cancer: a meta-analysis

**DOI:** 10.1186/1477-7819-10-172

**Published:** 2012-08-25

**Authors:** Jie Qin, Bo Xie, Qiqi Mao, Debo Kong, Yiwei Lin, Xiangyi Zheng

**Affiliations:** 1 Department of Urology, First Affiliated Hospital, Zhejiang University School of Medicine, Hangzhou, 310003, Zhejiang Province, China; 2 Department of Urology, Tongde Hospital of Zhejiang Province, Hangzhou, 310003, Zhejiang Province, China

**Keywords:** Green tea, Black tea, Bladder neoplasms, Meta-analysis

## Abstract

**Background:**

Tea consumption has been reported to be associated with an decreased risk of several types of cancers. However, the results based on epidemiological studies on the association of tea consumption with bladder cancer were inconsistent. This meta-analysis was undertaken to evaluate the relationship between tea consumption and bladder cancer risk.

**Methods:**

Eligible studies were retrieved via both computer searches and review of references. The summary relative risk (RR) with 95% confidence interval (CI) was calculated.

**Results:**

Twenty three studies met the inclusion criteria of the meta-analysis. No association with bladder cancer was observed in either overall tea consumption group (OR =0.94, 95% CI 0.85-1.04) or subgroups stratified by sex, study design, geographical region or tea types.

**Conclusions:**

Our findings did not support that tea consumption was related to the decreased risk of bladder cancer.

## Background

In the United States, an estimated 70,530 new cases of bladder cancer will be diagnosed and approximately 14,680 deaths were attributed to bladder cancer in 2010 [1]. Bladder cancer is the most expensive cancer to survey and treat because of the need for frequent interval cystourethroscopy, urine cytology and radiological evaluations [2]. Therefore, more and more attention has been given to chemoprevention. Cancer chemoprevention is defined as the use of natural, synthetic, or biologic chemical agents to reverse, suppress, or prevent carcinogenic progression to invasive cancer. Bladder cancer has a protracted course of progression and may be ideal for chemoprevention strategies [3].

Tea, derived from the plant Camellia sinensis, is one of the most common beverages consumed worldwide, especially in China. Multiple lines of evidence support a protective effect of tea on various cancers [4]. The emperor of China, ShenNung, is credited with first describing the therapeutic effects of tea in 2737 BC [5]. Studies conducted on cell-culture systems and animal models show that tea or the active ingredient in tea, polyphenols, could afford protection against a variety of cancer types [4]. However, the results based on epidemiological studies on the association of tea consumption with bladder cancer were inconsistent. A meta-analysis conducted in 2001 suggested the consumption of tea seems not to be related to an increased risk of urinary tract cancer [6].

The purpose of the present study was to update and quantitatively assess the association between tea consumption and the risk of bladder cancer by summarizing the results of published cohort and case-control studies. We also sought to address the unresolved issue of whether this relationship differs across the tea type.

## Results

In total, we identified 35 papers examining the risk of bladder cancer with tea consumption published between 1966 and December 2011, and these were reviewed by 2 authors. We found no relevant non-English language papers in this field. Six studies were excluded because of insufficient information to compute its relative risk (RR) and 95% confidence interval (CI) [7-9], or a summary odds ratio (OR) adjusted for at least age, sex and smoking [10-12]. Six studies were excluded because they were found to be subsets of other studies or have overlapping data and were excluded [13-18]. Thus, 23 studies [19-41] were included in the meta-analysis on the association of tea consumption with bladder cancer risk. There were six cohorts [26,29,31,33,38,40] and seventeen case–control studies; nine of these were population-based [19-21,23,27,30,34,37,41] and eight were hospital-based case-control studies [22,24,25,28,32,35,36,39]. Of the twenty-three studies, seventeen were conducted in Western countries [17,20-27,29,30,32-34,36,37,40,41], and six were conducted in Asia [19,28,31,35,38,39]. Only eight articles reported association between consumption of specific tea types (green or black tea) and the risk of bladder cancer [19,23,25,26,31,35,38,39]. Information on tea consumption was obtained by interview, self-administered questionnaire or both techniques.

Table 1 presents the basic characteristics of each study included in our meta-analysis. Of the seventeen case-control studies, most studies found no significant association between tea consumption and bladder cancer, whereas four studies reported significantly increased risks [20,28,36,41], and three found inverse associations [19,30,35]. Of the six cohort studies, five reported no significant association between tea consumption and bladder cancer [26,29,31,38,40], whereas one found significantly decreased risk [33].

**Table 1:** Study characteristics of published cohort and case-control studies on tea consumption and bladder cancer

**Authors and year**	**Study design**	**Country**	**Study period**	**Cases/ subjects**	**Anatomical site of urinary tract**	**Tea type**	**Variables of adjustment**	**Tea drinking assessment**
Ros *et al*. [40]	Cohort	European countries	1992-2000	513/233,236	Urinary tract	Tea	Age, sex, smoking status	Questionnaire
Hemelt *et al*. [39]	HCC	China	2005-2008	419/384 (green tea)	Bladder	Green tea	Age, sex, smoking status, smoking frequency, and smoking duration adjusted odds ratios	Questionnaire
				408/385 (black tea)		Black tea		
Kurahashi *et al*. [38]	Cohort	Japan	1990-2005	164/49,566 (men)	Bladder	Green tea	Age, area, smoking status, alcohol and coffee consumption	Questionnaire
				42/54,874 (women)				
Jiang *et al*. [37]	PCC	US	1987-1999	1,586/1,586	Bladder	Tea	Age, sex, race, level of education, use of NSAIDs, carotenoid intake, number of years as hairdresser/barber, cigarette smoking status, duration of smoking, and intensity of smoking.	Questionnaire
Stefani *et al*. [36]	HCC	Uruguay	1996-2000	255/501	Bladder	Tea	Age, sex, residence, urban/rural status, education, family history of bladder cancer among first-degree relatives, body mass index, occupation, smoking status, years since quitting, number of cigarettes smoked per day, maté drinking, soft drink intake, milk intake, and, coffee drinking	Both
Wakai *et al*. [35]	HCC	Japan	1994-2000	124/744	Urinary tract	Green tea	Age, sex, cumulative consumption of cigarettes, year of first visit	Questionnaire
						Black tea		
Woolcott *et al*. [34]	PCC	Canada	1992-1994	927/2118	Bladder	Tea	Age, sex, education level, current smoking, cumulative smoking, and intake of energy, calcium, fibre and beer	Questionnaire
Zeegers *et al*. [33]	Cohort	Netherland	1986-1992	569/3,123	Bladder	Tea	Age, sex, number of cigarettes/day, years of cigarette smoking, and coffee consumption	Questionnaire
Geoffery-Perez *et al*. [32]	HCC	France	1984-1987	765/765	Bladder	Tea	Age, sex smoking, residence, center	Interview
Bianch *et al*. [30]	PCC	USA	1986-1989	1,452/2,434	Bladder	Tea	Age, sex, education, smoking status, family history of bladder cancer, high risk occupation, total beverage consumption, years of chlorinated surface water, vegetable and coffee consumption	Questionnaire
Nagano *et al*. [31]	Cohort	Japan	1979-1981	114/3,8540	Bladder	Green tea	Age, gender, radiation dose, smoking status, education level, body-mass index, and calendar time	Questionnaire
						Black tea		
Lu *et al*. [28]	HCC	Taiwan	1996-1997	40/160	Bladder	Tea	age, sex, date of admission, family history, ethnicity, and smoking status.	Questionnaire
Michaud *et al*. [29]	Cohort	US	1986-1996	252/47,909	Bladder	Tea	Geographic region, age, pack-years of smoking, current smoking status, energy intake, intake of fruits and vegetables, and intake of all other beverages.	Questionnaire
Bruemmer *et al*. [27]	PCC	US	1987-1990	262/405	Bladder	Tea	Age, sex, smoking, county	Interview
Chyou *et al*. [26]	Cohort	US	1965-1968	96/7,995	Urinary tract	Green tea	Age, smoking	Both
						Black tea		
Kunze *et al*. [25]	HCC	Germany	1977-1985	675/675	Urinary tract	Black tea	Age, sex, smoking	Interview
D’Avanzo *et al*. [24]	HCC	Italy	1985-1990	555/855	Bladder	Tea	Age, sex, education, smoking habits, alcohol drinking and exposure to occupational risk	Interview
Nomura *et al*. [23]	PCC	US	1977-1986	261/522	Urinary tract	Black tea	Age, sex, pack-years of cigarette smoking	Interview
Clavel *et al*. [22]	HCC	France	1984-1987	690/690	Bladder	Tea	Age, sex, smoking	Interview
Slattery *et al*. [18]	PCC	US	1977-1982	419/889	Bladder	Tea	Age, sex, smoking status, diabetes and bladder infection	Interview
Risch *et al*. [21]	PCC	Canada	1979-1982	876/1,668	Bladder	Tea	Age, sex, residence, and lifetime cigarette consumption.	Interview
Jensen *et al*. [20]	PCC	Denmark	1979-1981	371/771	Bladder	Tea	Age, sex, smoking status,	Interview
Ohno *et al*. [19]	PCC	Japan	1976-1978	293/589	Urinary tract	Black tea	Age, smoking	Interview

PCC: population-based case-control study, HCC: hospital-based case-control study, NSAIDs: non-steroidal anti-inflammatory drugs, Both: questionnaire and interview.

**Figure 1:**
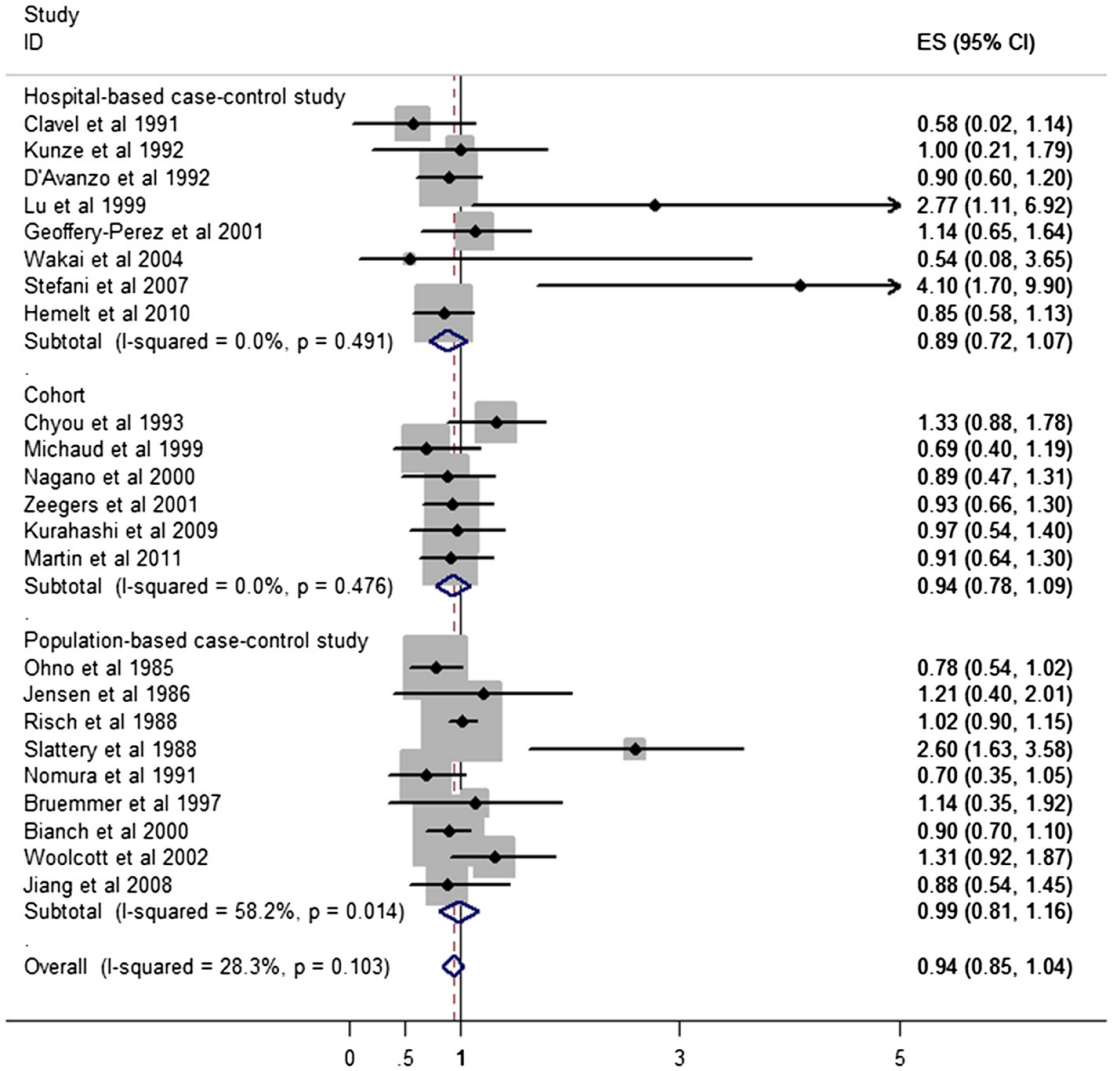
A forest plot showing risk estimates from case-control and cohort studies estimating the association between tea consumption and risk for bladder cancer.

**Figure 2:**
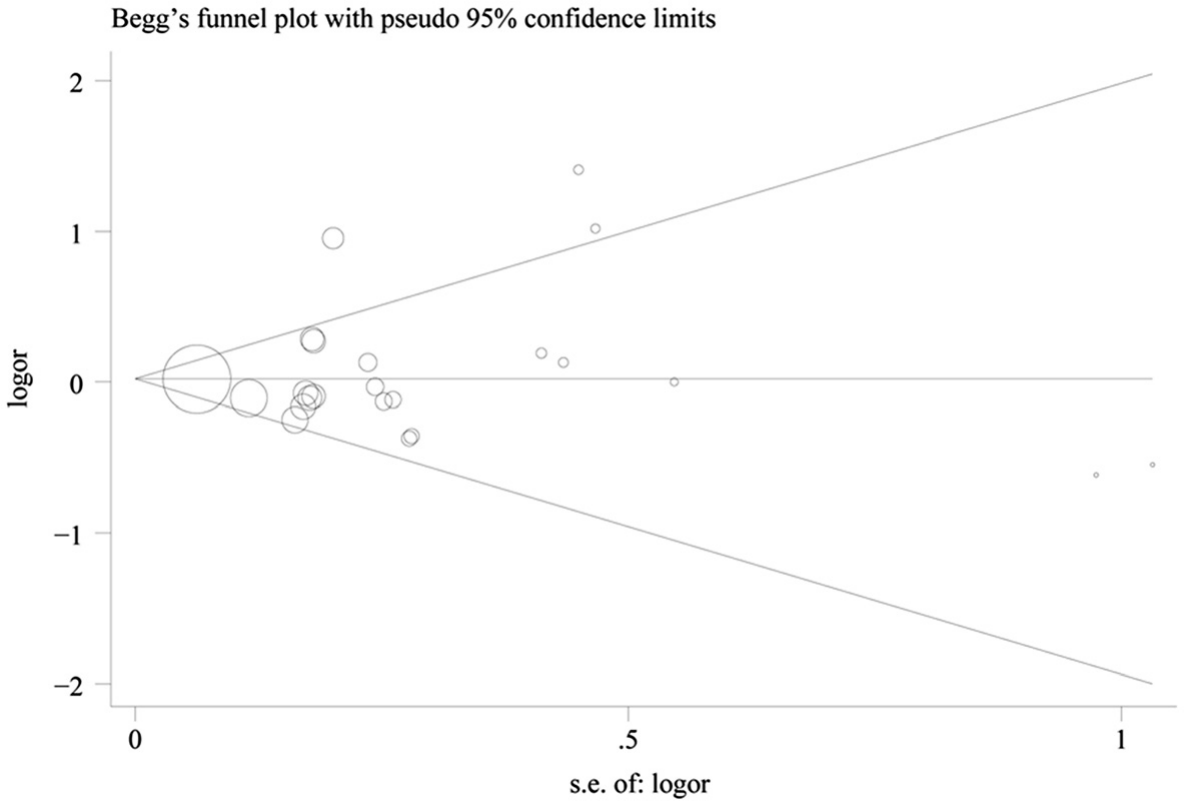
Funnel plot of tea consumption and bladder cancer risk.

Figure 1 plots the pooled risk estimates for overall tea consumption by study design. When all these studies were analyzed together, no association was observed for tea consumption with bladder cancer (OR 0.94, 95% CI 0.85, 1.04) and the summary ORs were similar across study design and source of the controls in case-control studies. No statistically significant heterogeneity was observed when all the studies were analyzed together. However, we noted some heterogeneity in the population-based case-control studies (I^2^ 58.2%, *P* = 0.014). After excluding one study by Slattery *et al*. [41], which reported the highest point estimates, the *P*-value for heterogeneity in the subgroup was no longer statistically significant (I^2^ 15.2%, *P* 0.311), and the summary OR was not significantly changed (OR 0.94, 95% CI 0.83, 1.05). There was no indication of publication bias from the Begg funnel plot (Figure 2).

**Figure 3:**
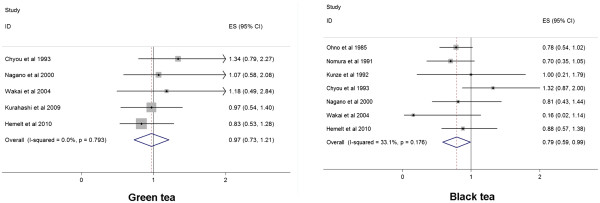
Forest plots showing the risk estimates from each study and the pooled risk estimates for green tea and black tea.

The results of subgroup analyzes by sex (men and women), geographical region (US/Canada, Europe, Uruguay and Asia) and tea type (green tea and black tea) are shown in Table 2. The OR estimates from subgroup analysis varied little, showing tea consumption was not associated with the likelihood of bladder cancer when separately analyzed by sex, geographical regions or tea type. In the subgroup analysis by tea type (Figure 3), we noted that green tea or black tea consumption was not associated with bladder cancer risk (OR 0.97, 95% CI 0.73, 1.21; OR 0.79, 95% CI 0.59, 0.99). There was weak heterogeneity among studies for black tea. We performed a sensitivity analysis, which removed one study at a time. This analysis confirmed the stability of the results for black tea. No indication of publication bias was observed from either with the Egger or Begg test in any subgroup.

**Table 2:** Summary of pooled odds ratios (ORs)for bladder cancer by sex, geographical region, and tea type

**Subgroup**	**Number of studies**	**Pooled OR**	**Q-test for heterogeneity**	**Egger test**	**Begg test**
		**(95% CI)**	***P*-value (I**^**2**^**score)**	***P*-value**	***P*-value**
All studies	23	0.94 (0.85, 1.04)	0.103 (28.3%)	0.518	0.267
Sex					
Men	10	1.03 (0.91, 1.14)	0.534 (0.0%)	0.446	0.348
Women	9	0.85 (0.69, 1.01)	0.461 (0.0%)	0.638	0.348
Geographical region					
Asia	6	0.85 (0.69, 1.00)	0.784 (0.0%)	0.360	0.348
Europe	7	0.92 (0.77, 1.08)	0.841 (0.0%)	0.789	0.881
Uruguay	1	4.10 (0.00, 8.20)	-	-	-
US/Canada	9	1.02 (0.83, 1.21)	0.008 (61.2%)	0.723	0.835
Tea type					
Green tea	5	0.97 (0.73, 1.21)	0.793 (0.0%)	0.377	0.221
Black tea	7	0.79 (0.59, 0.79)	0.176 (33.1%)	0.381	0.764

## Discussion

There has been considerable interest in the possible impact of tea consumption on bladder cancer risk due to the modifiable nature of tea consumption. In this meta-analysis of epidemiological studies of the association between tea and risk of bladder cancer including six cohorts and seventeen case-control studies, we found that tea consumption was not associated with reduced risk of bladder cancer.

A previous meta-analysis by Zeegers *et al*. [6] found no association between tea consumption and bladder cancer risk. The authors reported that the consumption of tea seems not to be related to an increased risk of urinary tract cancer. Our finding is consistent with their results. However, our study included 11 studies published after 2000 that were not included in the previous meta-analysis. We pooled the OR estimates by sex (men and women), tea type (green tea and black tea), geographical region (US/Canada, Europe, Uruguay and Asia), and study design (cohort or case-control studies).

Studies conducted on cell-culture systems and animal models as well as human epidemiological studies show that tea could afford protection against a variety of cancer types. Several laboratory studies have tried to investigate the link between tea and bladder cancer. Most of tea research on bladder cancer to date has focused on the effect and mechanism of green tea. It is generally agreed that many of the chemoprevention effects of green tea are mediated by polyphenols. The major catechins in green tea are epigallocatechin-3-gallate (EGCG), epicatechin-3-gallate, epigallocatechin, and epicatechin. EGCG accounts for 50% to 80% of catechin in green tea. Our previous study indicates that EGCG induces apoptosis in human bladder cancer T24 cells [42]. Kemberling *et al*. found that green tea (15% of which are polyphenols) have shown efficacy against rat bladder cancer induced by N-(4-hydroxybutyl)-N-bytyl-nitrosame (OH-BBN) [5]. The non-significant findings regarding the effects of tea consumption on bladder cancer in our meta-analyses contradict the results of previous experimental studies on this topic using *in vitro* bladder cancer cell lines and *in vivo* animal models. The difference between the results from experimental studies and our meta-analyses is likely to be due to the lower quantities of human tea consumption compared to the doses used in experimental studies and the fact that bioavailability is an important factor for consideration.

As a meta-analysis of previously published observational studies, our study has several limitations that need to be taken into account when considering its contributions. First, our meta-analysis only included published articles. Unpublished studies were not searched for our analysis. Second, we did not include studies with insufficient information to estimate an adjusted OR, which could bring publication bias even though no significant evidence of publication bias was observed in the Egger or the Begg tests. Third, our meta-analysis is likely affected by some misclassification of tea consumption. Tea exposure is mostly assessed regarding the number of cups of tea consumed daily or weekly. However, cup size may vary considerably. Fourth, only eight studies performed analyses and reported the RR separately for black tea and green tea. All other studies referred simply to tea. Finally, studies included in our meta-analysis were mainly conducted in Europe, US, Canada, Uruguay, and Asia; therefore, we are not able to generalize our findings for all populations. Also, most studies just included bladder cancer without specifying the type. Transitional cell carcinoma is the most common type of cancer in these regions and we found no relevant papers on squamous cell carcinoma. So the results are mainly based on transitional cell carcinoma but not squamous cell carcinoma.

## Conclusions

In conclusion, in this pooled analysis of six cohort and seventeen case-control studies, we did not find that tea consumption was associated with decreased risk of bladder cancer. Given the small number of cohort studies included in this meta-analysis, further research from large epidemiological studies is needed in this area.

## Methods

### Literature research

We searched and reviewed the MEDLINE database using PubMed, Web of Science and the Cochrane Library, using selected common key words related to tea consumption and bladder cancer risk in case-control and cohort studies. We also scanned bibliographies of relevant articles in order to identify additional studies. As the key words for the literature search, we selected tea for the exposure factors, and bladder cancer for the outcome factors. The articles evaluating the relationship of urinary tract cancer and tea consumption were also retrieved, because the overwhelming majority of tumors occurred in the bladder, and the renal pelvis and ureter are covered by the same urothelium. The term bladder cancer was used as a synonym for these neoplasms.

Each identified publication was reviewed and included in the analysis if all the following criteria were met: first, they had to be case-control or cohort studies; second, papers reported in English between 1966 and December 2011; last, the result of each study was expressed as relative risk (RR) or odds ratio (OR) together with its corresponding 95% confidence interval (95% CI) adjusted for age, sex and smoking at the least, or sufficient information allowing us to compute them.

### Data extraction

Data from all articles were retrieved independently by JQ and QM while the methods and results sections were removed and coded to blind the assessors to this information. The following data were collected: the first author’s name, the year of publication, country of origin, the study design (cohort or case-control), number of participants (cases and cohort size, or cases and controls), anatomical site of the neoplasm, adjusted effects estimates, exposure assessment and adjusted covariates. Considering that bladder cancer is a rare disease, the RR was assumed to be approximately the same as the OR, and the OR was used as the study outcome. Adjusted ORs were extracted directly from the original reports. If studies reported sex-stratified age- and smoking-adjusted ORs, we calculated the overall age-, smoking- and sex-adjusted OR by combining these estimates using the method of Mantel and Haenszel [43].

### Statistical analysis

We pooled data using the DerSimonian and Laird random effects models [44], which considers both within-study and between-study variation. Subgroup analyses were performed according to sex (male or female), study design (cohort or case-control studies), the study location (US/Canada, Europe, Uruguay or Asia) and tea types (green tea, black tea). We quantified the extent of heterogeneity using the Q-test [44] and the I^2^ score [45], and *P* < 0.05 was considered statistically significant. Publication bias was assessed using the tests of Egger [46] and Begg [47]. All statistical analyzes were performed using Stata Statistical Software, version 10.0.

## Abbreviations

RR: relative risk; CI: confidence interval; OR: odds ratio; NSAID: non-steroidal anti-inflammatory drug; EGCG: epigallocatechin-3-gallate; OH-BBN: N-(4-hydroxybutyl)-N-bytyl-nitrosame.

## Competing interests

The authors declare that they have no competing interests.

## Authors’ contributions

XZ and JQ conceived the study concept and participated in its design, data extraction, statistical analysis, manuscript drafting and editing. JQ and BX participated in the literature research, manuscript drafting and editing. QM participated in design and data extraction. DK participated in manuscript drafting, editing and statistical analysis. YWL conceived the study concept and participated in data analysis. All authors read and approved the final manuscript.
